# The p53/miRNAs/Ccna2 pathway serves as a novel regulator of cellular senescence: Complement of the canonical p53/p21 pathway

**DOI:** 10.1111/acel.12918

**Published:** 2019-03-07

**Authors:** Shun Xu, Weijia Wu, Haijiao Huang, Ruxiao Huang, Luoyijun Xie, Ailing Su, Shuang Liu, Ruinian Zheng, Yuan Yuan, Hui‐ling Zheng, Xuerong Sun, Xing‐dong Xiong, Xinguang Liu

**Affiliations:** ^1^ Institute of Aging Research Guangdong Medical University Dongguan China; ^2^ Guangdong Provincial Key Laboratory of Medical Molecular Diagnostics Guangdong Medical University Dongguan China; ^3^ The Scientific Research Center of Dongguan Guangdong Medical University Dongguan China; ^4^ Department of Oncology Dongguan People's Hospital Dongguan China; ^5^ Institute of Biochemistry & Molecular Biology Guangdong Medical University Zhanjiang China

**Keywords:** aging, Ccna2, cellular senescence, miRNA, p53

## Abstract

Aging is a multifactorial process characterized by the progressive deterioration of physiological functions. Among the multiple molecular mechanisms, microRNAs (miRNAs) have increasingly been implicated in the regulation of Aging process. However, the contribution of miRNAs to physiological Aging and the underlying mechanisms remain elusive. We herein performed high‐throughput analysis using miRNA and mRNA microarray in the physiological Aging mouse, attempted to deepen into the understanding of the effects of miRNAs on Aging process at the “network” level. The data showed that various p53 responsive miRNAs, including miR‐124, miR‐34a and miR‐29a/b/c, were up‐regulated in Aging mouse compared with that in Young mouse. Further investigation unraveled that similar as miR‐34a and miR‐29, miR‐124 significantly promoted cellular senescence. As expected, mRNA microarray and gene co‐expression network analysis unveiled that the most down‐regulated mRNAs were enriched in the regulatory pathways of cell proliferation. Fascinatingly, among these down‐regulated mRNAs, Ccna2 stood out as a common target of several p53 responsive miRNAs (miR‐124 and miR‐29), which functioned as the antagonist of p21 in cell cycle regulation. Silencing of Ccna2 remarkably triggered the cellular senescence, while Ccna2 overexpression delayed cellular senescence and significantly reversed the senescence‐induction effect of miR‐124 and miR‐29. Moreover, these p53 responsive miRNAs were significantly up‐regulated during the senescence process of p21‐deficient cells; overexpression of p53 responsive miRNAs or knockdown of Ccna2 evidently accelerated the cellular senescence in the absence of p21. Taken together, our data suggested that the p53/miRNAs/Ccna2 pathway might serve as a novel senescence modulator independent of p53/p21 pathway.

## 1 INTRODUCTION

Aging process is driven by various contributing factors, which inevitably impairs tissue function and increases susceptibility to disease and death (Lopez‐Otin, Blasco, Partridge, Serrano, & Kroemer, 2013). It is demonstrated that the presence and progressive accumulation of senescent cells contribute to overall organism Aging; senescent cells aggregate in Aging tissues have been considered as a causal factor for Aging‐related disorders (van Deursen, 2014; Smith‐Vikos & Slack, 2012). miRNAs have been well established to be critical regulators of gene expression in posttranscriptional level and thus are involved in multiple biological processes (Bartel, 2004). The emerging role of miRNAs in modulating Aging processes has gained increasing attention since the discovery of miRNAs that regulate lifespan in *Caenorhabditis elegans* (Boehm & Slack, 2005; Jung & Suh, 2012).

Senescent cells are characterized as irreversible growth arrest, increased senescence‐associated β‐galactosidase activity, and undergo distinctive phenotypic alterations, including profound chromatin and secretome changes (Campisi, 2005; van Deursen, 2014). Research over last three decades has uncovered a variety of signaling pathways that are involved in the regulation of cellular senescence and determine the lifespan in a manner conserved across species, including insulin growth factor 1 (IGF‐1) signaling (IIS), rapamycin (mTOR) signaling, and the sirtuin family (Delaney et al., 2011; Mazucanti et al., 2015). Additionally, p53 activation exerts critical roles in modulating cellular senescence and organismal Aging (Beausejour et al., 2003; Rufini, Tucci, Celardo, & Melino, 2013). Senescence‐induction stressors including DNA lesions, telomere shortening, oxidative stress, and oncogene activation, initially halt cell cycle progression through p53‐mediated induction of p21 and finally trigger cellular senescence.

MicroRNAs (miRNAs) are conserved tiny noncoding RNAs (18–25 nt in length) generated from endogenous hairpin‐shaped precursors, which have emerged as novel and fundamental actors in the gene regulation scenario (Flynt & Lai, 2008). These small RNA molecules can direct bind to specific sites presented in the 3'UTR of target mRNA, leading to either mRNA decay or translational blockade by formation of the RNA‐induced silencing complex (RISC; Bartel, 2004). As the recognition of target mRNAs mainly depends on the seed region of the mature miRNA, one single miRNA might regulate hundreds of target mRNAs; meanwhile, distinct miRNAs might co‐regulated the same mRNA, thus orchestrating a large variety of physiological and cellular processes (Bushati & Cohen, 2007).

Recently, a growing body of evidence has suggested the potential role of miRNAs in modulating the Aging process and cellular senescence (Felekkis, 2016). Nonetheless, the relationship between miRNAs and physiological mammalian Aging, and the detailed mechanism is still far to be elucidated (Williams, Smith, Kumar, Vijayan, & Reddy, 2017). In this work, we evaluated the miRNA and mRNA profile in the physiological Aging mouse model (20‐month C57 mouse) by high‐throughput analysis, sought to identify novel Aging‐related miRNAs, and further shed light on the roles of the effects of miRNAs on Aging process and illuminate the underlying molecular mechanisms.

## 2 RESULTS

### 2.1 Identification of Aging‐related miRNAs

**Figure 1:**
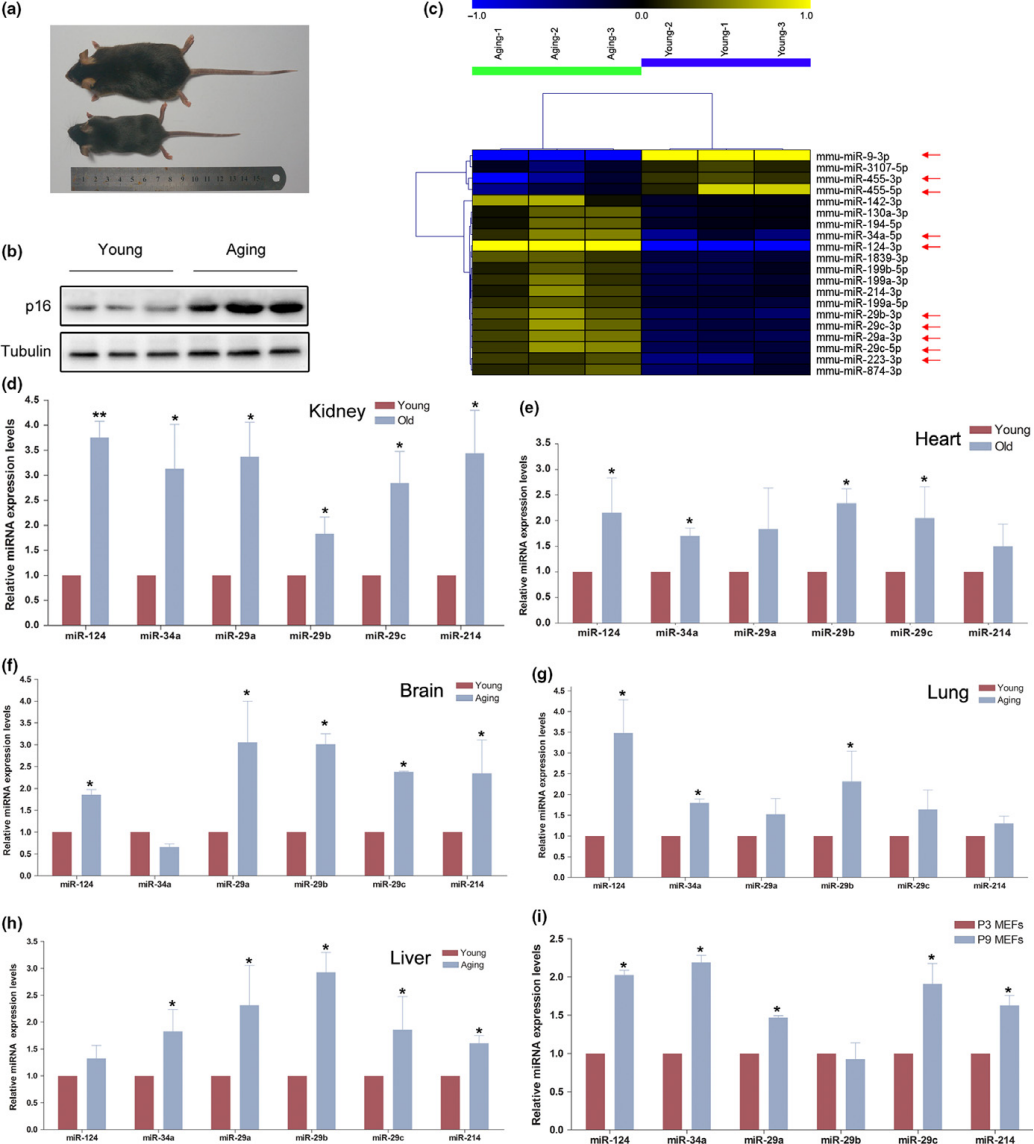
miR‐124, miR‐34a, and miR‐29a/b/c were significantly up‐regulated during Aging process. (a) Representative photographs of 2‐month‐old male C57BL/6 mouse and 20‐month‐old male C57BL/6 mouse. (b) The expression p16 levels in the kidney from 2‐month‐old male C57BL/6 mouse and 20‐month‐old male C57BL/6 mouse. (c) The most differentially expressed miRNAs in the kidney from 2‐month‐old male C57BL/6 mouse and 20‐month‐old male C57BL/6 mouse using miRNA microarray (*n* = 3). The color is determined by the ratio between the miRNA signal value of 20‐month and 2‐month mouse kidney. (d–h) The expression levels of miRNAs in the kidney, heart, brain, lung, and liver tissues from Aging mouse and Young mouse. Columns, mean of three independent experiments (*n* = 3); bars, *SEM*. *p < 0.05; **p < 0.01, compared to the Young mouse tissue. (i) The miRNA expression levels in P3 MEF and P9 MEF. Columns, mean of four independent experiments (*n* = 4); bars, *SEM*. *p < 0.05, comparison between two groups as indicated

To identify novel Aging‐related miRNAs, we initially established a physiological Aging mouse model (20‐month‐old male C57BL/6 mouse), compared with 2‐month‐old male C57BL/6 mouse (Figure 1a,b). Then, a miRNA microarray was performed to profile miRNA expression levels in kidney from 20‐month‐old male C57BL/6 mouse (designated as Aging) and 2‐month‐old male C57BL/6 mouse (designated as Young). We identified a total of 10 differentially expressed known miRNAs with ≥1.5 fold change (*p* < 0.05; Supporting Information Table S1, Figure 1c). In addition to several previously validated Aging‐related miRNAs (miR‐34a and miR‐29a/b/c), miR‐124 was the most up‐regulated miRNA in Aging mouse (Figure 1d, Supporting Information Table S1), which indicated the impact of miR‐124 on Aging process.

To confirm the validity of our screening technique, qPCR was performed to monitor the expression levels of miR‐124, miR‐34a, and miR‐29a/b/c in kidney, heart, liver, brain, and lung from Young and Aging mouse and verified the up‐regulation of these miRNAs in Aging mouse (Figure 1d–h). Moreover, we further established two typical cellular senescence models, including the replicative senescence model of MEFs through serial passage (P9), and H_2_O_2_‐induced senescence of NIH/3T3 cells. The premature senescence phenotype of P9 MEFs and H_2_O_2_‐treated NIH/3T3 cells was confirmed (Supporting Information Figure S1a,b). The results revealed that miR‐124, miR‐34a, and miR‐29a/b/c were notably up‐regulated in both cellular senescence models (Figure 1i and Supporting Information Figure S1c) as well. In addition, the expression levels of miR‐124, miR‐34a, and miR‐29a were examined in serum starvation‐induced quiescent MEFs. Different from the result in senescent MEFs, the expression levels of these miRNAs were down‐regulated in quiescent MEF cells (Supporting Information Figure S1d), which further indicated the specific association of miR‐124 with cellular senescence.

### 2.2 miR‐124 significantly promoted cellular senescence

**Figure 2:**
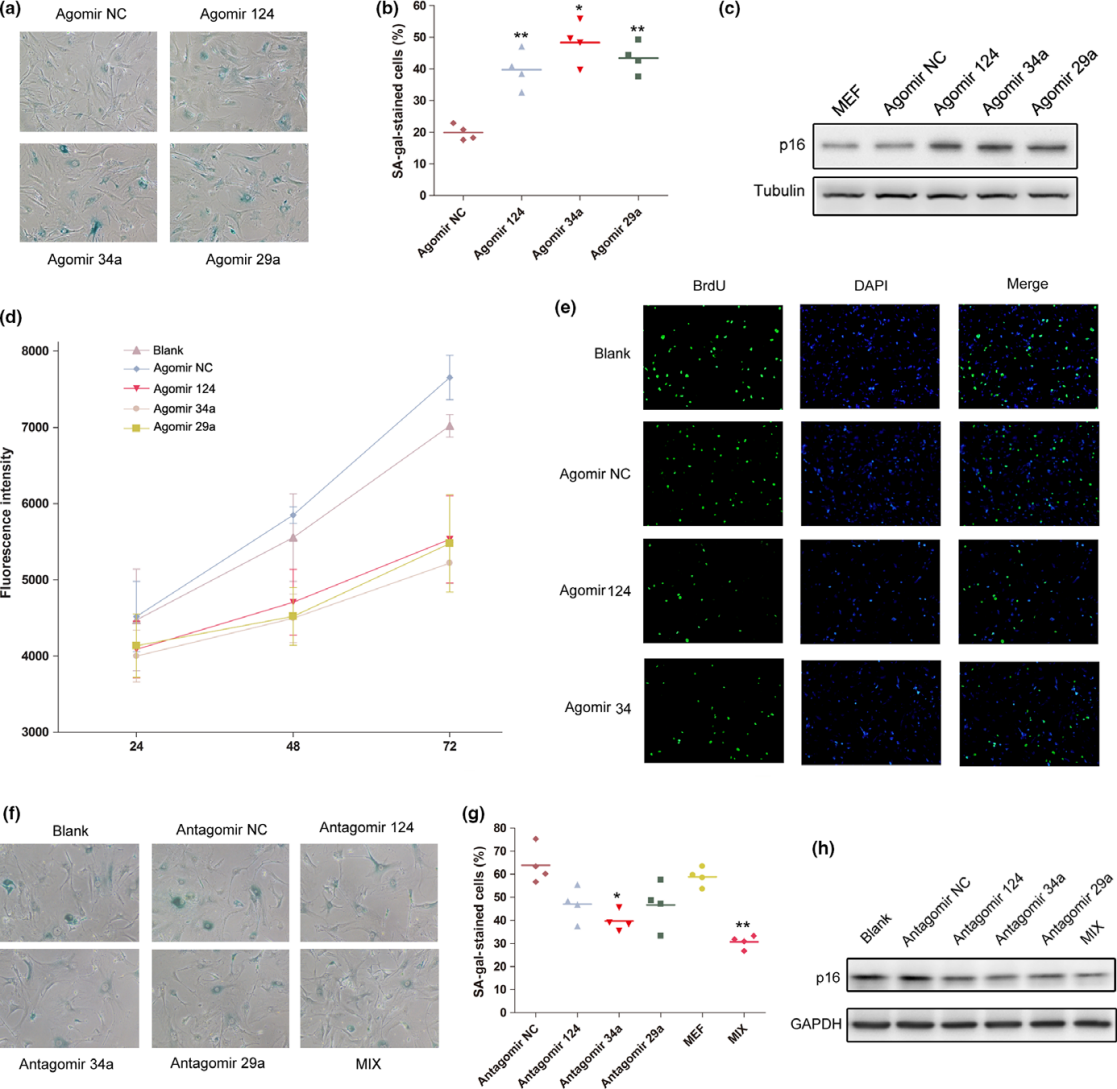
miR‐124 significantly promoted the replicative senescence of MEF cells. (a) Representative photographs of SA‐β‐gal staining of MEF cells transfected with Agomir NC, Agomir 124, Agomir 34a, and Agomir 29a. (b) The average SA‐β‐gal staining MEF cells transfected with Agomir NC, Agomir 124, Agomir 34a, and Agomir 29a. *p < 0.05; **p < 0.01, compared to the Agomir NC‐transfected MEFs. (c) The p16 expression in Agomir NC, Agomir 124, Agomir 34a, and Agomir 29a‐transfected MEFs. (d) Effect of miR‐124, miR‐34a, and miR‐29a on cell viability of MEFs transfected with Antagomir NC, Agomir 124, Agomir 34a, and Agomir 29a. (e) Representative images of cells stained with DAPI (blue fluorescence) and BrdU, as a measurement of DNA synthesis (green fluorescence) in Agomir NC, Agomir 124, Agomir 34a, and Agomir 29a‐transfected MEF cells. (f) Representative photographs of SA‐β‐gal staining of MEF cells transfected with miRNA inhibitors, including Antagomir NC, Antagomir 124, Antagomir 34a, Antagomir 29a, and all three inhibitors (MIX). (g) The average SA‐β‐gal staining MEF cells transfected with Antagomir NC, Antagomir 124, Antagomir 34a, Antagomir 29a, and MIX. *p < 0.05; **p < 0.01, compared to the Antagomir NC‐transfected MEFs. (h) The p16 expression in miRNA inhibitor‐transfected MEFs

To investigate the potential role of miR‐124 on cellular senescence, we overexpressed miR‐124, miR‐34a, and miR‐29a in MEFs by transfection MEFs with Agomir NC, Agomir 124, Agomir 34a, and Agomir 29a and assessed the subsequent effect of these miRNAs on MEFs senescence utilizing SA‐β‐gal staining assay and p16 evaluation by western blot. The result showed that MEFs transfected with Agomir 124 exhibited much more SA‐β‐gal‐positive cells than MEFs transfected with Agomir NC, similar as miR‐34a and miR‐29a (Figure 2a,b). Moreover, the p16 expression levels were evidently elevated in the MEFs transfected with Agomir 124, Agomir 34a, or Agomir 29a, which was consistent with the results of SA‐β‐gal staining (Figure 2c).

Furthermore, Alamar Blue assay and BrdU cell proliferation assay were carried out to measure the influence of miR‐124, miR‐34a, and miR‐29a on cell viability and proliferation of MEFs. The results showed that ectopic expression of miR‐124 endowed MEFs with relatively lower fluorescence activity (Figure 2d), and diminished BrdU incorporation (Figure 2f), similar as the effect of miR‐34a and miR‐29a on cell viability and proliferation, which was compatible with the senescence‐induction effect of these miRNAs.

Additionally, we transfected MEFs with miRNAs inhibitors, including Antagomir 124, Antagomir 34a, Antagomir 29a, or all three miRNA antagomirs (designated as MIX). The results of SA‐β‐gal staining and p16 detection showed that though inhibition of these three miRNAs separately only conferred modestly attenuated senescent phenotype in MEFs, whereas suppression of all three miRNAs in MEFs remarkably delayed the process of cellular senescence (Figure 2f–h). Moreover, we transfected agomirs or antagomirs into human cells—HUVECs. The results uncovered that these miRNAs significantly promoted H_2_O_2_‐induced HUVEC senescence similar as in MEFs (Supporting Information Figure S2a,b). Taken together, our data strongly suggested that miR‐124 facilitated the cellular senescence of MEFs similar as miR‐34a and miR‐29.

### 2.3 Identification of differentially expressed mRNAs in Aging process

**Figure 3:**
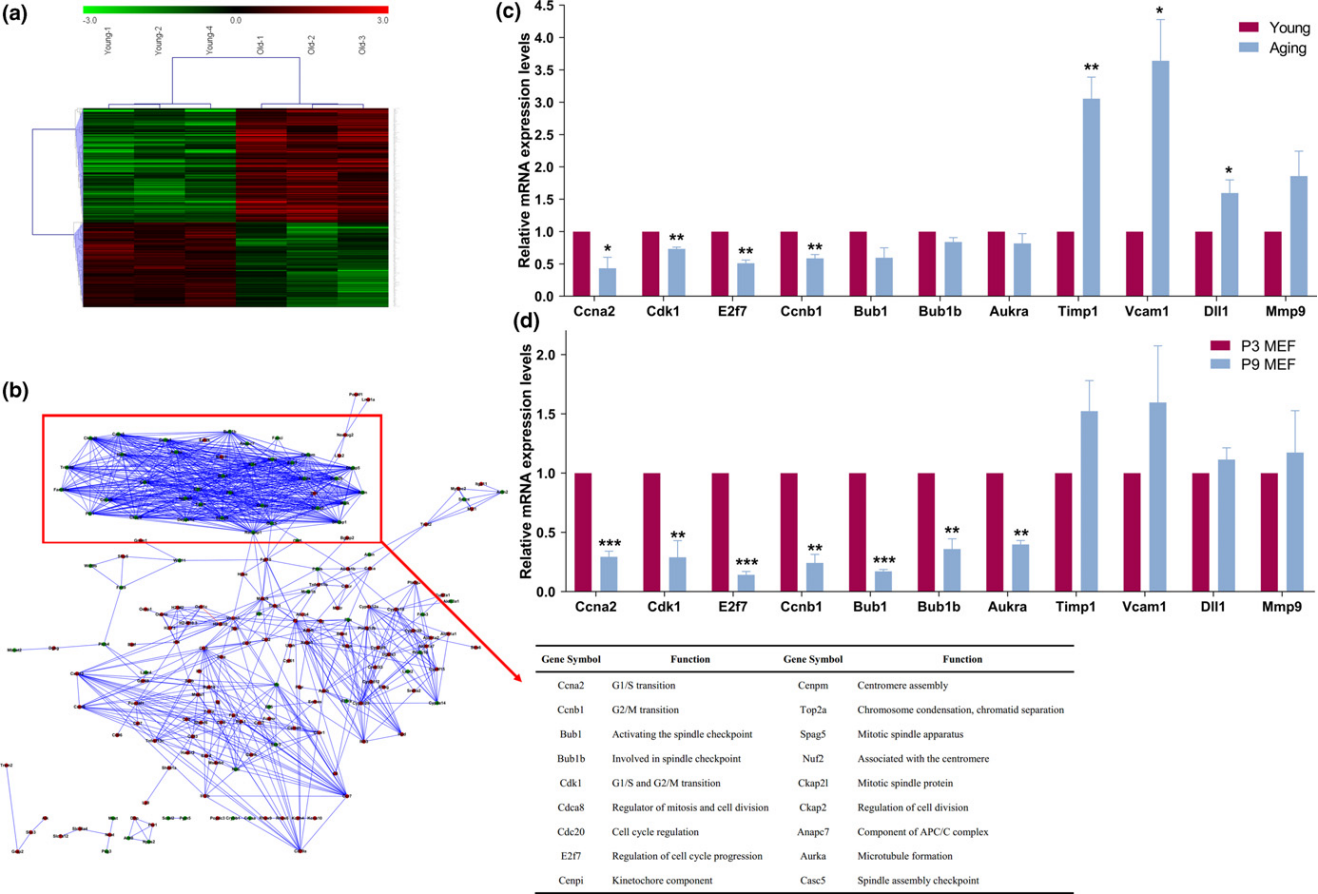
Identification differentially expressed mRNA during Aging process. (a) mRNA expression profile of 2‐month and 20‐month mouse kidney (*n* = 3) was evaluated by mRNA microarray. The heatmaps were generated from the hierarchical cluster analysis to show a distinguishable mRNA expression profile between Young and Aging mouse. The color is determined by the ratio between the mRNA signal value of 20‐month and 2‐month mouse kidney. (b) The gene co‐expression network analysis was performed to assess the association among the differentially expressed mRNAs. The gene symbol and function of the most down‐regulated mRNAs were listed. (c) The expression levels of partial mRNAs in the Aging and Young mouse kidney. Columns, mean of three independent experiments (*n* = 3); bars, *SEM*. *p < 0.05; **p < 0.01, compared to the Young mouse tissue. (d) The mRNA expression levels in P3 MEF and P9 MEF. Columns, mean of four independent experiments (*n* = 4); bars, *SEM*. **p < 0.01; ***p < 0.001, comparison between two groups as indicated

We further performed mRNA microarray in kidneys from Young (2‐month‐old C57BL/6 mouse) and Aging (20‐month‐old C57BL/6 mouse) group, in order to monitor the mRNA expression profile in Aging process. The data of mRNA microarray uncovered hundreds of differentially expressed mRNAs between Young and Aging mouse with a fold change ≥2.0 (*p* < 0.05; Figure 3a, Supporting Information Table S2). Then, a gene co‐expression network analysis was performed to evaluate the association among these differentially expressed mRNAs. Expectedly, the results unraveled that the most down‐regulated mRNAs were enriched in the regulatory pathways of cell proliferation, including Ccna2, Cdk1, E2f7, and Ccnb1 (Figure 3b), indicating the critical role of these mRNAs in Aging process. The down‐regulation of partial of these mRNAs was further confirmed by real‐time qPCR both in Aging mouse and in senescent MEFs (Figure 3c,d).

### 2.4 Ccna2 dramatically delayed the cellular senescence

**Figure 4:**
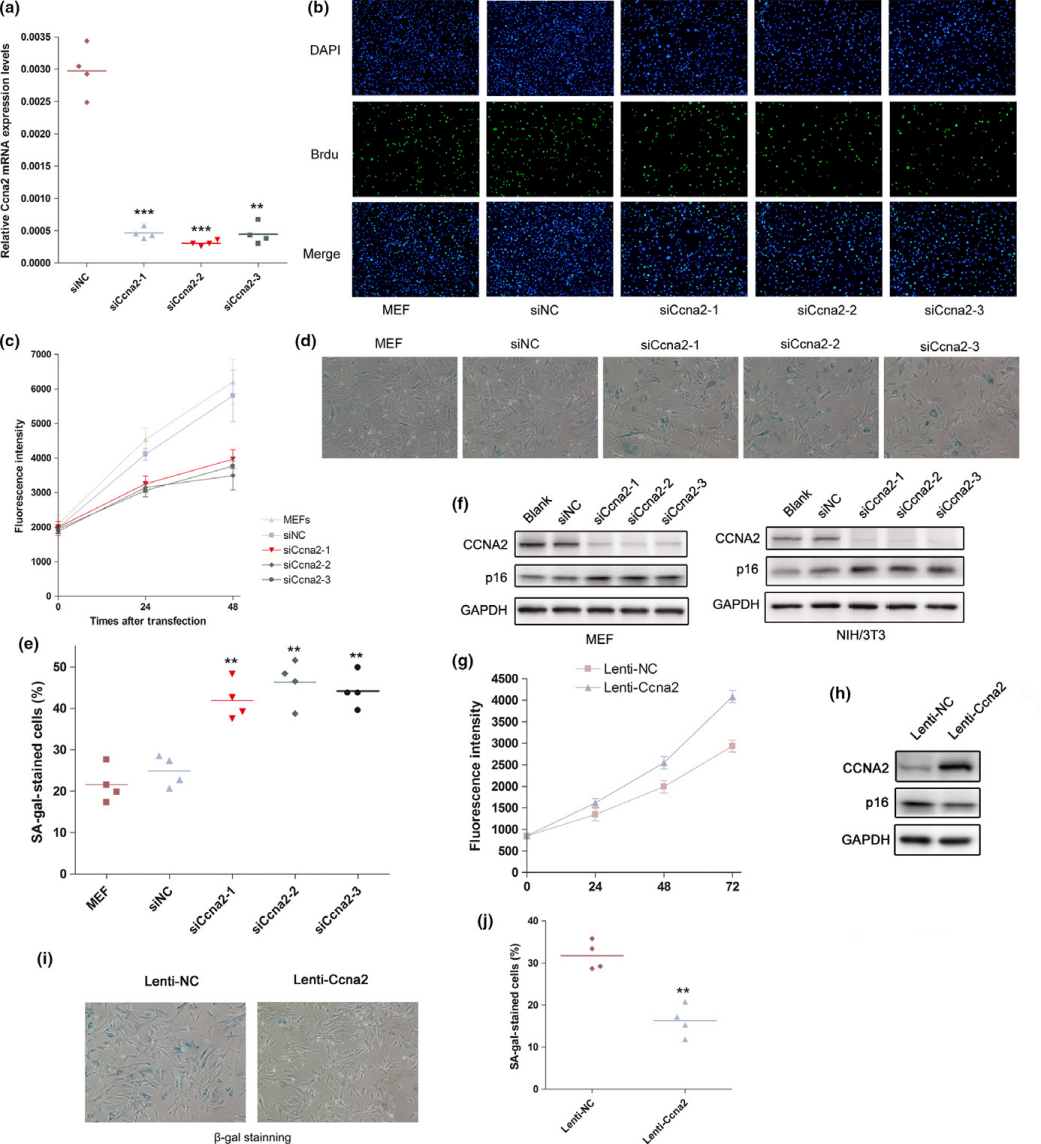
Ccna2 significantly delayed the cellular senescence. (a) The Ccna2 mRNA and protein levels in MEFs transfect with siNC and siCcna2‐1/2/3. (b) Representative images of cells stained with DAPI (blue fluorescence) and BrdU (green fluorescence) in siNC‐ and siCcna2‐1/2/3‐transfected MEF cells. (c) Effect of siCcna2 on cell viability of MEFs transfected with siNC and siCcna2‐1/2/3. (d) Representative photographs of SA‐β‐gal staining of MEF cells transfected with siNC and siCcna2‐1/2/3. (e). The average SA‐β‐gal staining MEF cells transfected with siNC and siCcna2‐1/2/3. **p < 0.01, compared with the blank and siNC‐transfected MEFs. (f) The p16 expression in siNC‐ and siCcna2‐1/2/3‐transfected MEFs (left) or NIH/3T3 cells (right). (g) Effect of Ccna2 overexpression on cell viability of MEFs infected with Lenti‐NC and Lenti‐Ccna2. (h) The p16 and Ccna2 protein expression in Lenti‐NC‐ and Lenti‐Ccna2‐infected MEFs. (i) Representative photographs of SA‐β‐gal staining of MEF cells infected with Lenti‐NC and Lenti‐Ccna2. (j) The average SA‐β‐gal staining MEF cells infected with Lenti‐NC and Lenti‐Ccna2. **p < 0.01, compared to the Lenti‐NC‐infected MEFs

In spite of the critical role of Ccna2 in regulation of cell cycle, the effect of Ccna2 on modulation of Aging process was still far to be elucidated. Thus, we specifically knocked down Ccna2 expression using designed siCcna2‐1/2/3. The inhibition of Ccna2 in MEF cells was confirmed (Figure 4a). Furthermore, the results of SA‐β‐gal staining assay and p16 evaluation showed that silencing of Ccna2 endowed MEFs with decreased cell viability and proliferation (Figure 4b,c), and enhanced premature senescence phenotype (Figure 4d–f). In addition, NIH/3T3 cells were transfected with siNC or siCcna2‐1/2/3; knockdown of Ccna2 significantly promoted the H_2_O_2_‐induced senescence (Supporting Information Figure S3c,d) and inhibited the cell viability and proliferation as well (Supporting Information Figure S3a,b).

Furthermore, we prepared a lentivirus containing the coding sequence of Ccna2 (designated as Lenti‐Ccna2); the overexpression of Ccna2 in MEF cells was confirmed (Figure 4h). The results revealed that ectopic expression of exogenous Ccna2 substantially delayed the replicative senescence (Figure 4h–j) and enhanced the cell viability of MEFs (Figure 4g). Thus, the above data demonstrated the important effect of Ccna2 on cellular senescence.

### 2.5 Ccna2 was a common target of p53 responsive miRNAs

**Figure 5:**
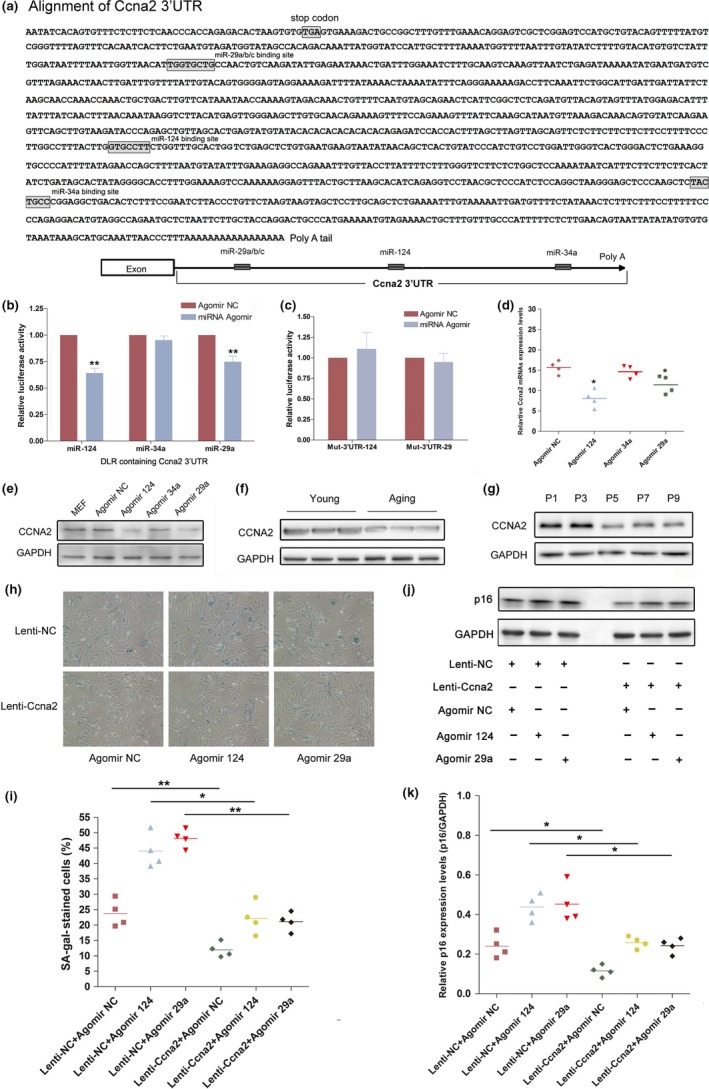
Ccna2 was the common target of miR‐124 and miR‐29. (a) Alignment and schematic diagram of Ccna2 3'UTR and the predicted miRNA binding sites. (b) Determination of luciferase activity. Cells were co‐transfected with Ccna2‐3'UTR with Agomir NC, Agomir 124, Agomir 34a, or Agomir 29a. Columns, mean of three independent experiments done in duplicate (*n* = 3); bars, *SEM*. **p < 0.01, compared with Agomir NC‐transfected cells. (c) Cells were co‐transfected with Mut‐3'UTR‐124 with Agomir NC or Agomir 124, or co‐transfected with Mut‐3'UTR‐29 with Agomir NC or Agomir 29a. Columns, mean of at least three independent experiments done in duplicate; bars, *SEM*. (d) Real‐time qPCR was used to monitor the Ccna2 expressions in MEF cells transfected with Agomir NC, Agomir 124, Agomir 34a, and Agomir 29a. *p < 0.05, comparison between two groups as indicated. (e) The Ccna2 protein expression levels in MEF cells transfected with Agomir NC, Agomir 124, Agomir 34a, and Agomir 29a. (f and g). Ccna2 protein expression levels in Aging mouse (f) and senescent MEF cells (g). (h and i) Representative photographs (h) and average (i) of SA‐β‐gal staining of MEF cells infected with Lenti‐NC and Lenti‐Ccna2 after transfected with Agomir 124, Agomir 29a, or Agomir NC. *p < 0.05, **p < 0.01, comparison between two groups as indicated. (j and k) The p16 protein expression and the intensity ratio between p16 and GAPDH in MEF cells infected with Lenti‐NC and Lenti‐Ccna2 after transfected with Agomir 124, Agomir 29a, or Agomir NC. *p < 0.05, comparison between two groups as indicated

To probe into the underlying molecular mechanisms by which these Aging‐related miRNAs modulate the Aging process, we predicted the putative targets of miR‐124, miR‐34a, and miR‐29 utilizing 3 pure algorithm prediction software, including miRWalk (http://zmf.umm.uni-heidelberg.de/apps/zmf/mirwalk2/), miRMap (http://mirmap.ezlab.org/), and TargetScan (http://www.targetscan.org/). To obtain more genuine target genes, we then compared the down‐regulated mRNAs in the mRNA microarray assays (inverse correlated with the expression of up‐regulated miRNAs) with the predicted target genes. Interestingly, among the generated miRNA–target pairs, Ccna2, one of the pivotal regulator of cell cycle, had a perfect match with the “seed region” of these Aging‐related miRNAs (Figure 5a, Supporting Information Table S3), indicating that Ccna2 might be the common target of these miRNAs.

To verify the direct binding of Ccna2 3'UTR with these Aging‐related miRNAs, we constructed a dual‐luciferase reporter system by cloning the mouse Ccna2‐3'UTR fragment (containing the three predicted binding sites of miR‐124, miR‐29, and miR‐34a) downstream of the renilla luciferase reporter in psi‐Check2. Subsequently, 293T cells were co‐transfected with the constructed vectors and Agomir NC, Agomir 124, Agomir 34a, or Agomir 29a. As a result, miR‐124 and miR‐29a significantly diminished the relative luciferase activity of the constructed vectors containing Ccna2‐3'UTR, whereas miR‐34a had no significant effect (Figure 5b). We further constructed two luciferase reporter vectors carrying mutant sequence in the complementary site for the seed region of miR‐124 or miR‐29a in Ccna2‐3'UTR (designated as Mut‐3'UTR‐124 or Mut‐3'UTR‐29). The results showed that mutation of miR‐124 or miR‐29a binding site in the Ccna2 3'UTR significantly reversed the inhibition of luciferase reporter activity by miR‐124 or miR‐29a (Figure 5c). Moreover, we evaluated the Ccna2 protein expression levels in MEFs after transfection with Agomir NC, Agomir 124, Agomir 34a, or Agomir 29a. miR‐124 and miR‐29a but not miR‐34a significantly suppressed the expression of endogenous Ccna2 expression levels (Figure 5d,e). Additionally, the Ccna2 protein expression levels were notably down‐regulated in Aging mouse and senescent MEF cells (Figure 5f,g).

Furthermore, MEFs were infected with Lenti‐Ccna2 (without miRNA binding sites) or control lentivirus (Lenti‐NC) after transfected with Agomir 124, Agomir 29a, or Agomir NC. SA‐β‐gal staining uncovered that ectopic expression of exogenous Ccna2 significantly reversed the senescence‐induction effect of Agomir 124 and Agomir 29a in MEFs (Figure 5h,i). In all, these data strongly suggested that Ccna2 was the common target of miR‐124 and miR‐29.

### 2.6 p53/miRNAs/Ccna2 was an independent pathway of p53/p21 pathway

Previous evidence has established that p53 activation is the key event during Aging process; p53 initially halts cell cycle progression through induction of p21 in response to various stressors and finally triggers cellular senescence. Attractively, we noted that multiple Aging‐related miRNAs (including miR‐124 and miR‐29) almost belong to p53 responsive miRNAs (Jeong et al., 2015; Liao, Cao, Zhou, & Lu, 2014). And the pri‐miRNA expression levels of miR‐124, miR‐34a, and miR‐29 were dramatically up‐regulated in senescent MEFs (Supporting Information Figure S4a), indicating that the up‐regulation of these miRNAs was mainly due to the transcriptional modulation. To further validate the relationship between p53 activity and these miRNA expression levels, MEFs were treated with pifithrin‐α (PFTα, p53 inhibitor) and tenovin‐1 (p53 activator). Tenovin‐1 treatment significantly enhanced the pri‐miRNAs and mature miRNAs of miR‐124, miR‐34a, and miR‐29 expression, while PFTα evidently suppressed the expression of the pri‐miRNAs and mature miRNAs of these miRNAs (Supporting Information Figure S4b–d). In addition, silencing of p53 in MEFs using sip53 significantly inhibited the pri‐miRNAs of these miRNAs (Supporting Information Figure S4e–g).

**Figure 6:**
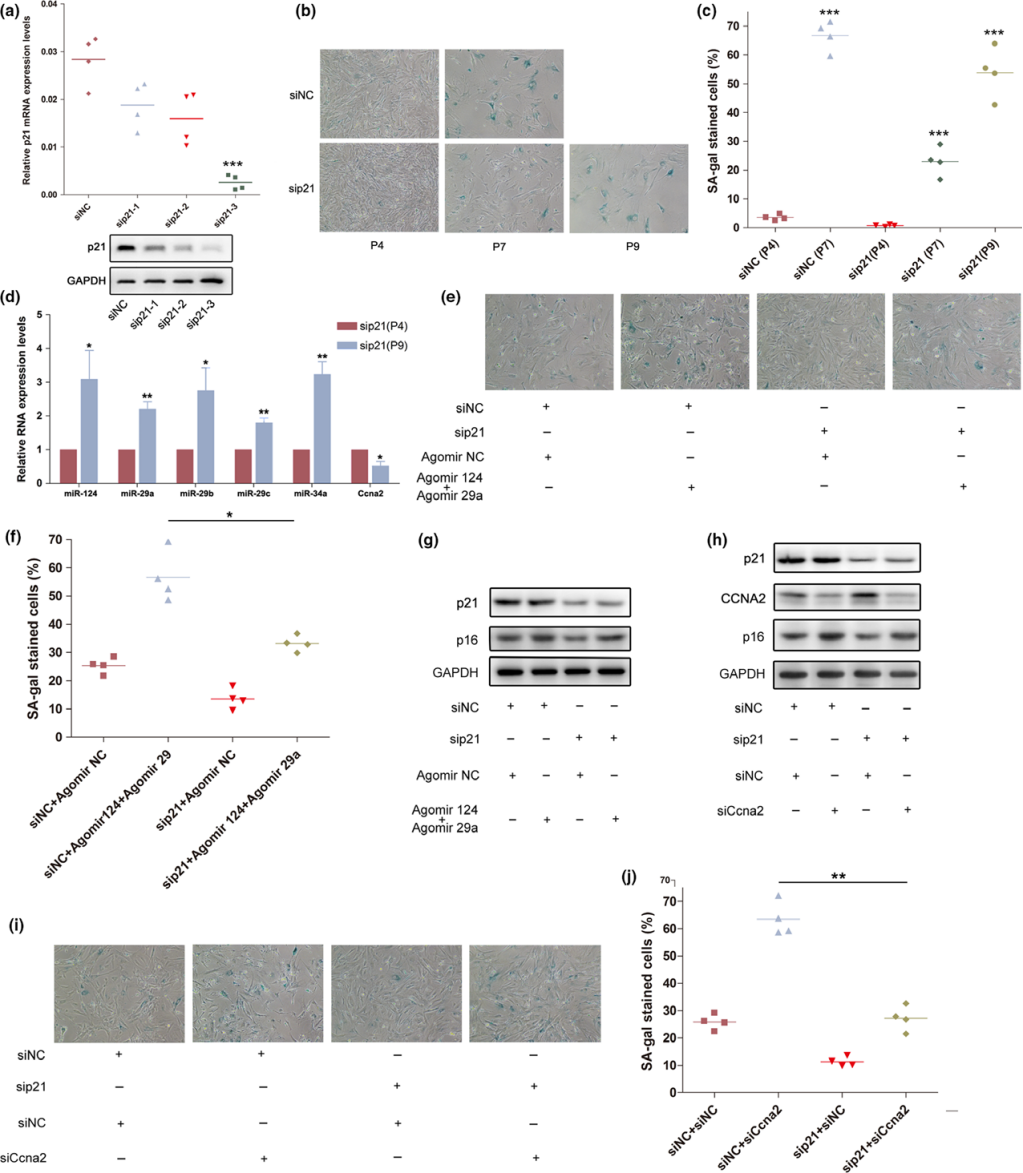
p53/p53 responsive miRNA/Ccna2 pathway served as a novel regulator of cellular senescence independent of p53/p21 pathway. (a) The p21 mRNA and protein levels in MEFs transfect with siNC, sip21‐1/2/3. (b) Representative photographs of SA‐β‐gal staining of MEF cells transfected with siNC and sip21 (sip21‐3). (c) The average SA‐β‐gal staining MEF cells transfected with siNC and sip21. ***p < 0.001, compared to the siNC‐transfected MEFs. (d) The miRNA expression levels in sip21‐transfected MEF cells. Columns, mean of four independent experiments (*n* = 3); bars, *SEM*. *p < 0.05; **p < 0.01, comparison between two groups as indicated. (e) Representative photographs of SA‐β‐gal staining of MEF cells co‐transfected with siNC or sip21, and Agomir 124/Agomir 29a or Agomir NC. (f) The average SA‐β‐gal staining MEF cells transfected with siNC or sip21, and Agomir 124/Agomir 29a or Agomir NC. *p < 0.05, compared to the siNC and Agomir 124/Agomir 29a‐transfected MEFs. (g) The p16 and p21 protein expression of MEF cells co‐transfected with siNC or sip21, and Agomir 124/Agomir 29a or Agomir NC were evaluated by western blot. (h) The p16, p21, and CCNA2 protein expression of MEF cells co‐transfected with siNC or sip21, and siNC or siCcna2 (siCcna2‐2). (i) Representative photographs of SA‐β‐gal staining of MEF cells co‐transfected with siNC or sip21, and siNC or siCcna2. (j) The average SA‐β‐gal staining MEF cells transfected with siNC or sip21, and siNC or siCcna2. **p < 0.01, compared to the siNC‐ and siCcna2‐transfected MEFs

Moreover, we noticed that Ccna2 competes with p21 to direct bind and activate Cdk2 and thus promotes G1/S transition. Hence, we speculated that, in addition to the induction of p21, p53 activation might notably enhance the expression of several Aging‐related miRNAs, which direct target Ccna2, the antagonist of p21, leading to cell cycle arrest, and finally give rise to cellular senescence. To deepen into the relationship between p53/p21 pathway and the potential p53/miRNAs/Ccna2 pathway, we knocked down p21 in MEFs utilizing designed sip21. The silencing of endogenous p21 by sip21 was confirmed (Figure 6a). Interestingly, though knockdown of p21 significantly delayed cellular senescence, sip21‐transfected MEF cells still exhibited premature senescence phenotype after serial passage (sip21 transfection was performed in each passage; Figure 6b,c). And during the senescence process of sip21‐transfected MEF cells, these Aging‐related p53 responsive miRNAs were notably up‐regulated in senescent p21‐deficient cells (P9) than that in Young p21‐deficient cells (P4; Figure 6d).

Additionally, we transfected Agomir 124 and Agomir 29a or Agomir NC into p21‐deficient cells. SA‐β‐gal staining showed that overexpression of these miRNAs notably promoted MEF senescence in the absence of p21 (Figure 6e–g). Moreover, we transfected siCcna2 or siNC into p21‐deficient MEF cells. The results unraveled that silencing of Ccna2 dramatically accelerated the senescence process of p21‐deficient MEF cells as well (Figure 6h–j).

We further knocked down the p21 expression in NIH/3T3 cells and observed that H_2_O_2_ could still induce the senescence phenotype in sip21‐transfected NIH/3T3 cells, accompanied with enhanced p53 responsive miRNA expression levels (Supporting Information Figure S5a,b). In addition, sip21‐transfected NIH/3T3 cells were transfected with Agomirs or siCcna2. Similar as in MEFs, ectopic expression of miR‐124 and miR‐29a or silencing of Ccna2 substantially promoted the H_2_O_2_‐induced senescence phenotype in NIH/3T3 cells in the absence of p21 (Supporting Information Figure S5c,d). In aggregate, the above results indicated the p53/miRNAs/Ccna2 pathway probably serves as an important regulator of cellular senescence, which was independent of p53/p21 pathway.

## 3 DISCUSSION

Accumulated evidences have established that the p53 and p53/p21 pathways exert critical effect during Aging process. We herein unveiled that there is another Aging‐related pathway downstream of p53—p53/p53 responsive miRNAs/Ccna2, in addition to the canonical p53/p21 pathway: p53 activation induces the expression of a handful of miRNAs, including miR‐124, miR‐29a/b/c, which target the p21 antagonist—Ccna2, and finally lead to cellular senescence. The discovery of the novel regulatory pathway further verified the pivotal role of p53 in Aging process and the close relationship between p53 responsive miRNAs and cellular senescence.

Previous studies have identified various p53 responsive miRNAs, including miR‐34a, miR‐29a/b/c, miR‐124, miR‐199a, and miR‐194 (Jeong et al., 2015; Liao et al., 2014). Among these miRNAs, miR‐34a and miR‐29 have been demonstrated to be typical Aging‐related miRNAs (Boon et al., 2013; Hu et al., 2014); in addition, miR‐194 was closely associated with cellular senescence as well (Xu et al., 2017). Though miR‐124 has been reported to be associated with several Aging‐related disease, including Alzheimer's disease and Parkinson's disease (Fang et al., 2012; Wang, Ye et al., 2016a), the effect and underlying mechanism of miR‐124 on Aging process were largely unknown. Our demonstration that miR‐124 functioned as an important regulator of cellular senescence through targeting Ccna2 further deepened the understanding about the central role of p53 responsive miRNAs during Aging process. Nevertheless, further investigations were still required to shed light on the effect of other p53 responsive miRNAs (such as miR‐199a) on Aging regulation.

In addition to the vital role of miR‐124 in neurogenesis (Papagiannakopoulos & Kosik, 2009), miR‐124 has been established to be a typical tumor suppressor in diverse human cancers, including lung cancer, prostate cancer, cholangiocarcinoma, and colorectal cancer (Wang, Chen et al., 2016b), and enhanced expression of miR‐124 significantly inhibited the cell proliferation of cancer cells (Tian et al., 2017; Zhou, Xu, Ren, Chen, & Xin, 2016), which was consistent with the anti‐proliferation and senescence‐induction function of miR‐124 in MEF cells in our study. On the contrary, another group has reported that miR‐124 was down‐regulated in wrn‐1 mutant mice and *C. elegans*, two accelerated Aging models, indicating an opposite effect of miR‐124 in the progeroid disorder Werner syndrome (Dallaire et al., 2012). We speculated that the effect of miR‐124 in wrn‐1 mutant‐accelerated Aging model might probably due to the neuroprotection effect, while the senescence‐induction effect of miR‐124 in physiological Aging model mainly through suppressing cell cycle. Nonetheless, the association between miR‐124 and Aging process still requires further investigations to fully elucidate this issue.

Cyclin A2 (Ccna2) exerts critical role in the control of G1/S transition through binding and activating Cdk2 (Blanchard, 2000), and loss of Ccna2 and Cdk2 dramatically impaired the cell proliferation (Gopinathan et al., 2014). In addition, Ccna2 has been reported to promote DNA damage repair (DSBs repair) in the brain during Aging process (Gygli et al., 2016). However, the effect of Ccna2 in Aging process was still elusive. Expectedly, our data uncovered Ccna2 were evidently down‐regulated in the Aging mouse tissues and senescent MEF cells, similar as multiple other cell cycle regulators, and silencing of Ccna2 significantly enhanced the premature senescence phenotype, which established a direct relationship between Ccna2 and cellular senescence. Our results further showed that Ccna2 was the common target of several Aging‐related miRNAs, including miR‐124 and miR‐29, which was consistent with the previous evidence that miR‐124 direct target Ccna2 (Das, Jana, & Bhattacharyya, 2013). What's more, the miRNA–target prediction analysis revealed that Ccna2 3'UTR contains candidate binding site with more other p53 responsive miRNAs (such as miR‐199a and miR‐194), indicating of the pivotal role of Ccna2 in the Aging‐related p53/miRNAs/Ccna2 pathway. Nonetheless, further investigations were still required to fully elucidate the relationship between Ccna2 and p53 responsive miRNAs.

In this study, we fully screened the miRNA and mRNA profile in the Aging mouse, uncovered various differentially expressed miRNAs and mRNAs, and further unraveled the senescence‐induction effect of miR‐124 through targeting Ccna2. Moreover, we presented evidences that the p21 antagonist, Ccna2, was the common target of several p53 responsive miRNAs, and the p53/miRNAs/Ccna2 pathway probably functioned as a novel regulator of Aging process independent of the typical p53/p21 pathway. Hence, we herein proposed a hypothesis that the two Aging‐related pathways downstream of p53 provided for the “Double Security,” which halt the cell cycle, and finally leading to cellular senescence (Supporting Information Figure S6).

## 4 EXPERIMENTAL PROCEDURES

### 4.1 Mouse tissues, MEFs preparation, and cell culture

C57BL/6 mice of male were sacrificed at 20 months (Aging) or 2 months (Young) old in the experiments; then, the kidney, lung, liver, heart, and brain tissues were obtained from both groups. The primary MEF cells were prepared from C57BL/6 mouse have been previously described (Xu et al., 2016) and considered as passage 0 (designated as P0). HK293T was a SV40‐transformed embryonic kidney cell line, and NIH/3T3 was an immortalized mouse embryonic fibroblasts. MEFs, HK293T, and NIH/3T3 were all cultured in DMEM supplemented with 10% FBS (Gibco, USA). Serial passage was performed when the cells reached 80%–90% confluence.

The study was approved by the Medical Ethics Committee of Guangdong Medical University. All animal experiments were performed in accordance with the ethical guidelines for animal protection rights in China.

### 4.2 RNA isolation and real‐time qPCR

Total RNA was extracted from MEFs, NIH/3T3, or mouse tissues using TRIzol reagent (Life Technologies, USA) according to the manufacturer's protocol. The quality and quantity of the extracted RNAs were determined from OD_260/280 nm_ reading by DS‐11 spectrophotometer (DeNovix, USA).

For miRNA, 1 μg total RNA was reverse transcribed using All‐in‐One miRNA qRT‐PCR Detection Kit (GeneCopoeia, USA) according to the manufacturer's recommendations. qRT‐PCR was then carried out in a LightCycler 96 (Roche, Switzerland) with All‐in‐One miRNA qRT‐PCR Detection Kit (GeneCopoeia, USA). Primers specific for miR‐124, miR‐34a, miR‐29a/b/c, miR‐214, miR‐194, miR‐199a, miR‐142, miR‐223, and U44 were all purchased from GeneCopoeia. U68 was included as internal control.

For mRNA, 1 μg total RNA was reverse transcribed using PrimeScript RT Reagent Kit with gDNA Eraser (Takara, Japan) according to the manufacturer's recommendations. qRT‐PCR was performed in a LightCycler 96 (Roche, Switzerland) with SYBR Select Master Mix (Life Technologies, USA). The primers used for Ccna2, Cdk1, E2f7, Ccnb1, Bub1, Bub1b, Aukra, Timp1, Vcam1, Dll1, Mmp9, and β‐Actin are listed in Supporting Information Table S4. β‐Actin gene was used as internal control.

### 4.3 miRNA microarray analysis

Microarray assays of miRNAs in Young (2 months) and Aging (20 months) old mouse kidney were performed by CapitalBio Corporation (Beijing, China). The extracted RNA was purified utilizing mirVanaTM miRNA Isolation Kit (Ambion, USA), in order to enrich the global miRNA, and then, the purified RNA was labeled and hybridized as described previously (Li et al., 2014). Briefly, the purified miRNAs were labeled using the Agilent miRNA labeling reagent (Agilent Technologies, USA), and the labeled miRNA was hybridized to the Agilent miRNA microarray designed with eight identical arrays per slide (8 × 60 K format), with each array containing probes interrogating 1,247 mouse miRNAs from the Sanger database (Version 19.0).

The miRNA array data were analyzed using the GeneSpring software V12 (Agilent Technologie). Microarray assay was performed in triplicates, utilizing three independent sets of RNA preparations. And differentially detected miRNA signals with ≥1.5 fold change and *p < *0.05 were considered significant. Finally, the hierarchical clustering was performed for the differentially expressed miRNAs using Cluster 3.0 and Java Treeview (Stanford University, USA).

### 4.4 mRNA microarray analysis

Gene expression microarray analysis in Young (2 months) and Aging (20 months) old mouse kidney was performed by CapitalBio Corporation (Beijing, China). Initially, double‐stranded cDNA (dsDNA) was synthesized from 100 ng of extracted RNA utilizing the CbcScript reverse transcriptase with cDNA synthesis system according to the manufacturer's instructions (CapitalBio) with the T7 Oligo (dT). Then, the amplified cRNA was synthesized from the obtained dsDNA using a T7 Enzyme Mix in vitro. Furthermore, 2 μg cRNA was reverse transcribed using the CbcScript II reverse transcriptase. The obtained cDNA products were purified and then labeled by Klenow enzyme labeling strategy. The labeled cDNA was hybridized to the Agilent mRNA Array designed with eight identical arrays per slide (8 × 60 K format), with each array containing probes interrogating about 39,430 Entrez Gene RNAs according to the manufacturer's recommendations.

The array data were analyzed using the GeneSpring software V12 (Agilent Technologie). Microarray assay was performed in triplicates, utilizing three independent sets of RNA preparations. And differentially detected mRNA signals with ≥2 fold change and *p *＜ 0.05 were considered significant. Finally, the hierarchical clustering was performed for the differentially expressed mRNAs using Cluster 3.0 and Java Treeview (Stanford University, USA).

### 4.5 Data availability

The miRNA and mRNA microarray data included in this study are available in the NCBI Gene Expression Omnibus (GEO). The GEO accession numbers are GSE106108 (miRNA) and GSE106109 (mRNA), respectively.

### 4.6 RNA oligoribonucleotides and cell transfections

The RNA duplex‐mimicked miR‐124, miR‐34a, and miR‐29a were designated as Agomir 124, Agomir 34a, and Agomir 29a. The control RNA duplex, designated as Agomir NC, was nonhomologous to any mouse genome sequences. The three inhibitors of miR‐124, miR‐34a, and miR‐29a were designated as Antagomir 124, Antagomir 34a, and Antagomir 29a, and the negative control was named as Antagomir NC. The sequence of the above RNA oligoribonucleotides was listed in Supporting Information Table S5 and was all purchased from GenePharma (China). In addition, the small interference RNAs (siRNA) targeting Ccna2 mRNA (GenBank accession no. NM_009828.3, designated as siCcna2) and p21 mRNA (GenBank accession no. NM_001111099.2, designated as sip21) were purchased from OriGene (USA).

Transfection of RNA oligoribonucleotide(s) was performed using Lipofectamine RNAiMAX (Life Technologies, USA) according to the manufacturer's instructions. For MEFs transfection, MEFs were plated in growth medium at a density of ∼60%–70% confluence (passage 3–4 for Agomir or siRNA transfection, and passage 5–6 for Antagomir transfection). 50 nM of Agomir or siRNA duplex or 50 nM Antagomir RNA was used for each transfection, unless otherwise indicated.

### 4.7 Cell viability assays

Cell viability was determined by the Alamar Blue assay (AbD Serotec, UK) as previously described (Xu et al., 2016). Briefly, MEFs were firstly seeded in a 96‐well plate at 50% confluence; 24 hr later, the cells were transfected with RNA oligoribonucleotide(s) and followed by the Alamar Blue assay at indicated times. Fluorescence of the Alamar blue dye was measured by Synergy 2 microplate fluorescence reader (BioTek, USA) at 540 nm excitation wavelength and 590 nm emission wavelength.

### 4.8 Senescence‐associated β‐galactosidase staining

Senescence‐associated β‐galactosidase staining (SA‐β‐gal) of cells transfected with indicated RNA oligoribonucleotides was performed utilizing the X‐gal staining kit (Sigma‐Aldrich, USA). Briefly, the cells were washed by PBS and fixed (2% formaldehyde, 0.2% glutaraldehyde in PBS). The fixed cells were then incubated with fresh β‐galactosidase staining solution at pH 5.8 and incubated at 37°C at least 12 hr. SA‐β‐galactosidase‐positive cells were detected by inverted bright field microscopy (Nikon, Japan) at 100× magnification.

### 4.9 BrdU incorporation assay

BrdU (5‐bromo‐2‐deoxyuridine) incorporation assay for measuring DNA synthesis (Sigma‐Aldrich, USA) was performed to evaluate the capability of cell proliferation. Briefly, MEFs (5 × 10^4^ cells per well) were cultured in triplicate in 24‐well plates and transfected with RNA oligoribonucleotide(s). Forty‐eight hours later, cells were incubated with 40 μM BrdU for an additional 1 hr at 37°C and then were fixed. The fixed cells were treated with 0.05% Trypsin to permeabilize cells, followed incubation with 3% BSA for 1 hr at room temperature or overnight at 4°C. Cells were further incubated with mouse anti‐BrdU monoclonal antibody (CST, USA), followed by Alexa Fluor 488‐conjugated secondary antibody (Molecular Probes, USA). Finally, cells were stained with DAPI (Sigma‐Aldrich, USA) as counterstain and read under a fluorescence microscope (Olympus, Japan).

### 4.10 Western blot

Initially, the cell protein lysates were separated on 10% or 12% SDS polyacrylamide gels, electrophoretically transferred to PVDF membranes (0.22 μm pore size; Millipore, USA). The transferred PVDF membrane was then incubated with rabbit anti‐p16 polyclonal antibody (10883‐1‐AP, Protein Tech, China), mouse anti‐CCNA2 monoclonal antibody (ab38, Abcam, USA), anti‐p53 polyclonal antibody (10442‐1‐AP, Protein Tech, China), or anti‐p21 polyclonal antibody (sc‐471, Santa Cruz Biotechnology, USA), followed by the respective secondary antibodies conjugated with horseradish peroxidase (HR) and subjected to a commercial enhanced chemiluminescence (ECL) kit (Pierce, USA). Protein loading was estimated using mouse anti‐tubulin monoclonal antibody (T5168, Sigma‐Aldrich, USA) or mouse anti‐GAPDH monoclonal antibody (60004‐1‐Ig, Protein Tech, China).

### 4.11 Vector construction

To construct the luciferase reporter vector (designated as Ccna2‐3'UTR), a wild‐type 3' UTR fragment of Ccna2 containing the putative binding sites for mR‐124, miR‐34a, and miR‐29 was amplified by PCR and then inserted downstream of the stop codon of Renilla luciferase in psi‐Check2 luciferase vector (Promega, USA). The mutant luciferase reporter vector (designated as Mut‐3'UTR‐124 or Mut‐3'UTR‐29) was constructed by inserting a 3'UTR fragment of Ccna2 carrying a mutated sequence in the binding site for miR‐124 or miR‐29 into psi‐Check2.

### 4.12 Lentivirus package

The lentivirus for Ccna2 overexpression (designated as Lenti‐Ccna2) was prepared utilizing the lentivirus package system purchased from Genepharma (China). Briefly, the recombinant lentiviral vector encoding Ccna2 (pGLV5‐Ccna2) and the two packaging plasmids pVSV‐G and pRev were co‐transfected into HEK‐293T cells at 80%–90% confluence. The cell culture medium was collected 48 hr after transfection, and the supernatant was then filtered through a 0.45‐μm filter. The lentivirus without the coding sequence of Ccna2 was used as the negative control (designated as Lenti‐NC).

### 4.13 Luciferase reporter assay

Briefly, 293T cells (4 × 10^4^cells per well) were plated in a 48‐well plate; 24 hr later, cells were co‐transfected with 10 nM either Agomir 124, Agomir 34a, Agomir 29a, or Agomir NC, and 100 ng of the constructed luciferase reporter vector using FuGENE™ HD Transfection Reagent (Promega, USA) according to the manufacturer's recommendations. Cells were collected using the Dual‐Luciferase Reporter Assay System 48 hr after transfection (Promega, USA). The dual‐luciferase activity was further determined by FB12 Luminometer (Berthold, Germany). Renilla luciferase activity of each sample was normalized by firefly luciferase activity.

### 4.14 Bioinformatics

The candidate target prediction of miR‐124, miR‐34a, and miR‐29 was performed using miRWalk (http://zmf.umm.uni-heidelberg.de/apps/zmf/mirwalk2/), miRMap (http://mirmap.ezlab.org/) and TargetScan (http://www.targetscan.org/). The gene co‐expression network analysis was performed by the STRING database (http://string-db.org).

### 4.15 Statistical analysis

Data are presented as mean ± SEM from at least three separate experiments. Unless otherwise noted, the differences between two groups were analyzed using Student's two‐tailed *t* test, while the differences between multiple groups were assessed by one‐way analysis of variance (ANOVA). *p* < 0.05 was considered statistically significant (table S6).

### 4.16 Accession number

GenBank Accession numbers for Ccna2 mRNA sequence (NM_009828.3) and p21 mRNA sequence (NM_001111099.2) are found at the National Center for Biotechnology Information (http://www.ncbi.nlm.nih.gov/). The sequences of miR‐124 (MIMAT0000134), miR‐34a (MI0000584), and miR‐29a (MI0000576) described in this study have been deposited in miRBase (http://www.mirbase.org/).

## CONFLICT OF INTEREST

None declared.

## AUTHOR CONTRIBUTIONS

Shun Xu, Weijia Wu and Haijiao Huang, Ruxiao Huang, Luoyijun Xie, and Ailing Su carried out the molecular experiments and the statistical analysis, and Shun Xu drafted the manuscript. Ruinian Zheng, Yuan Yuan, and Hui‐ling Zheng performed the animal experiments. Shun Xu, Xing‐dong Xiong, and Xinguang Liu participated in the design of the study. Xinguang Liu and Xing‐dong Xiong helped to revise the manuscript. All authors read and approved the final manuscript.
