# Supplementary figures and images for: The p53/miRNAs/Ccna2 pathway serves as a novel regulator of cellular senescence: Complement of the canonical p53/p21 pathway
Source: Aging Cell. 2019 Mar 7;18(3):e12918. doi: 10.1111/acel.12918 (PMC6516184; doi:10.1111/acel.12918)

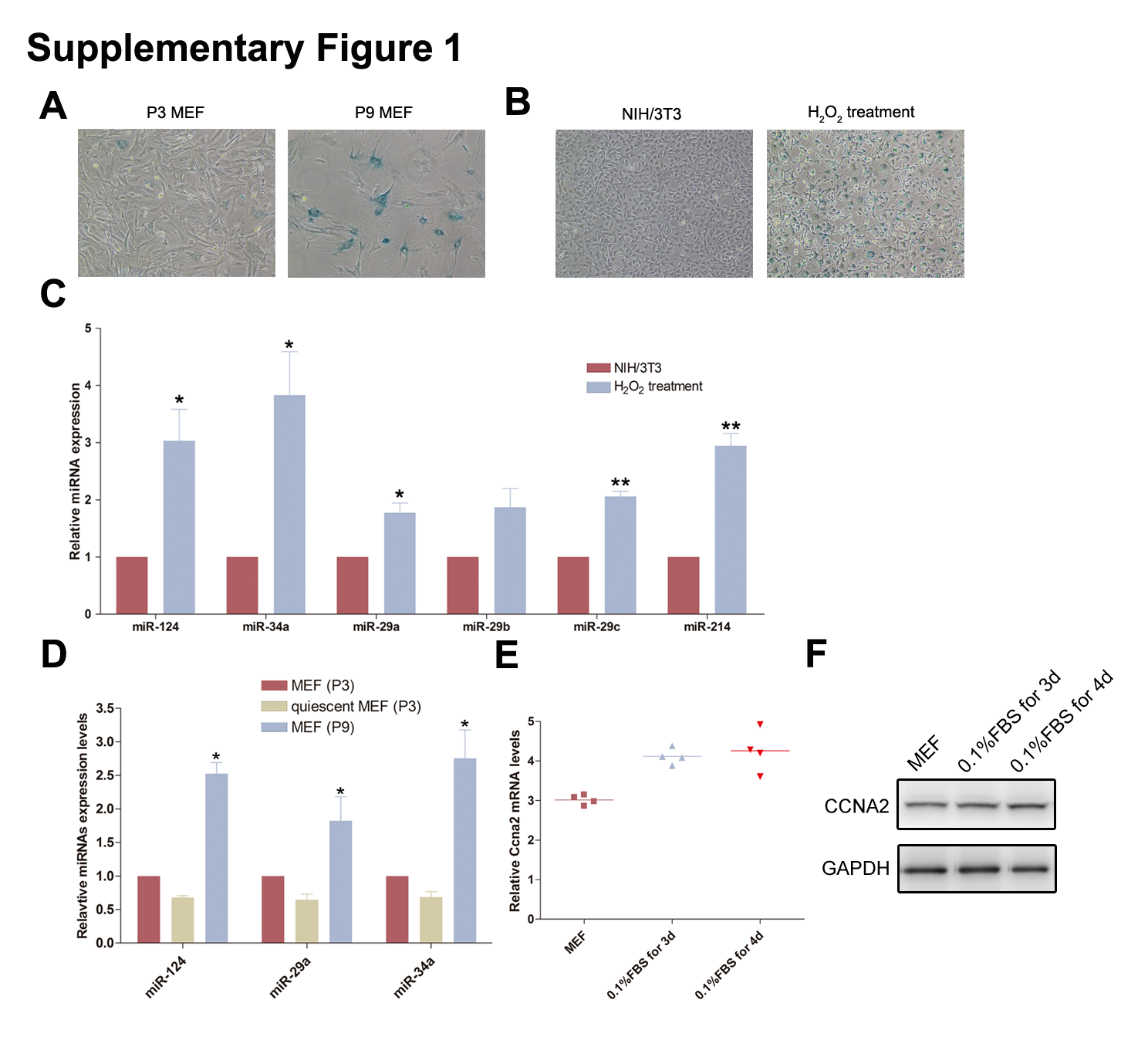

Supplement: Supplementary file 1 [file ACEL-18-e12918-s001.tif]

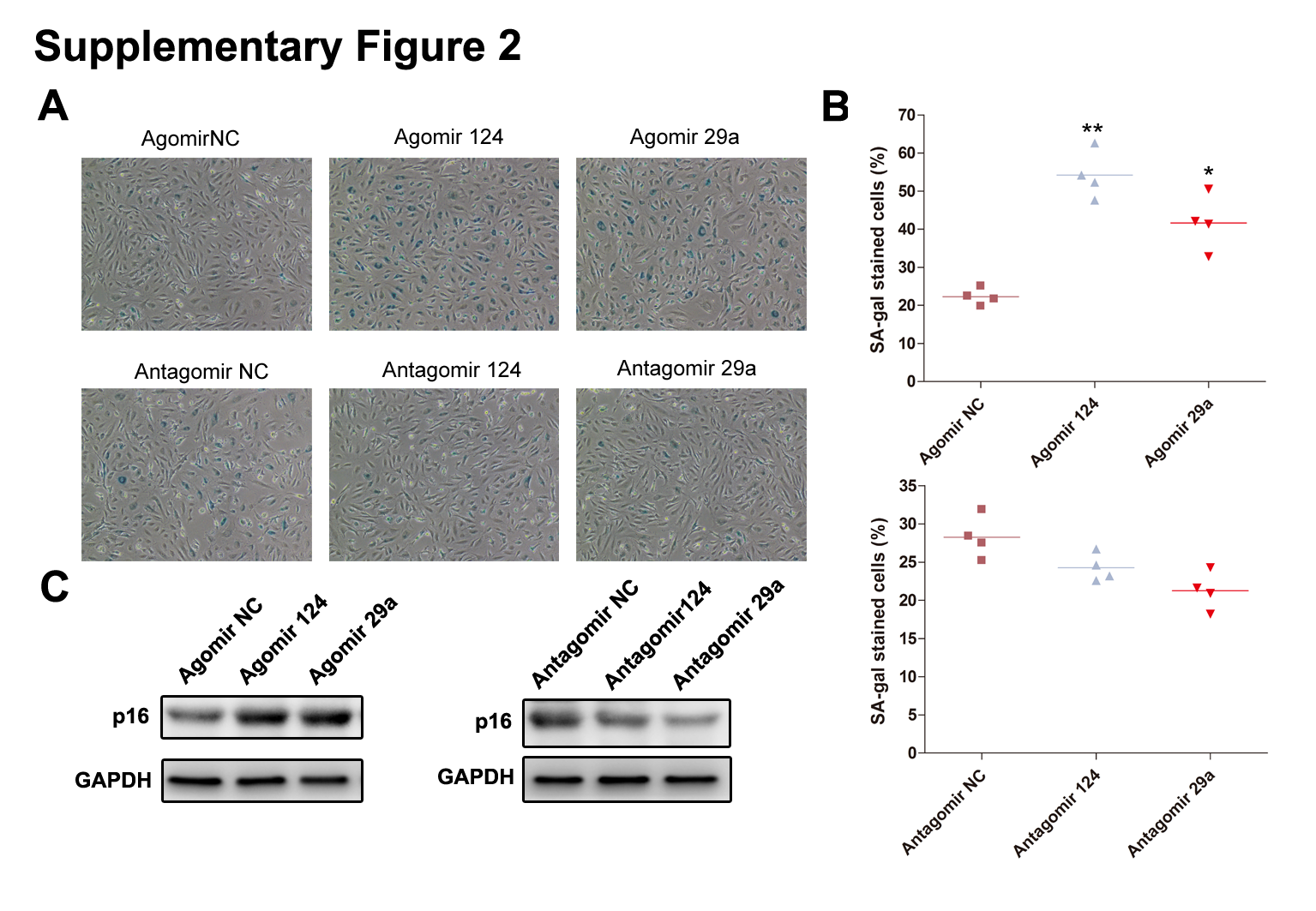

Supplement: Supplementary file 2 [file ACEL-18-e12918-s002.tif]

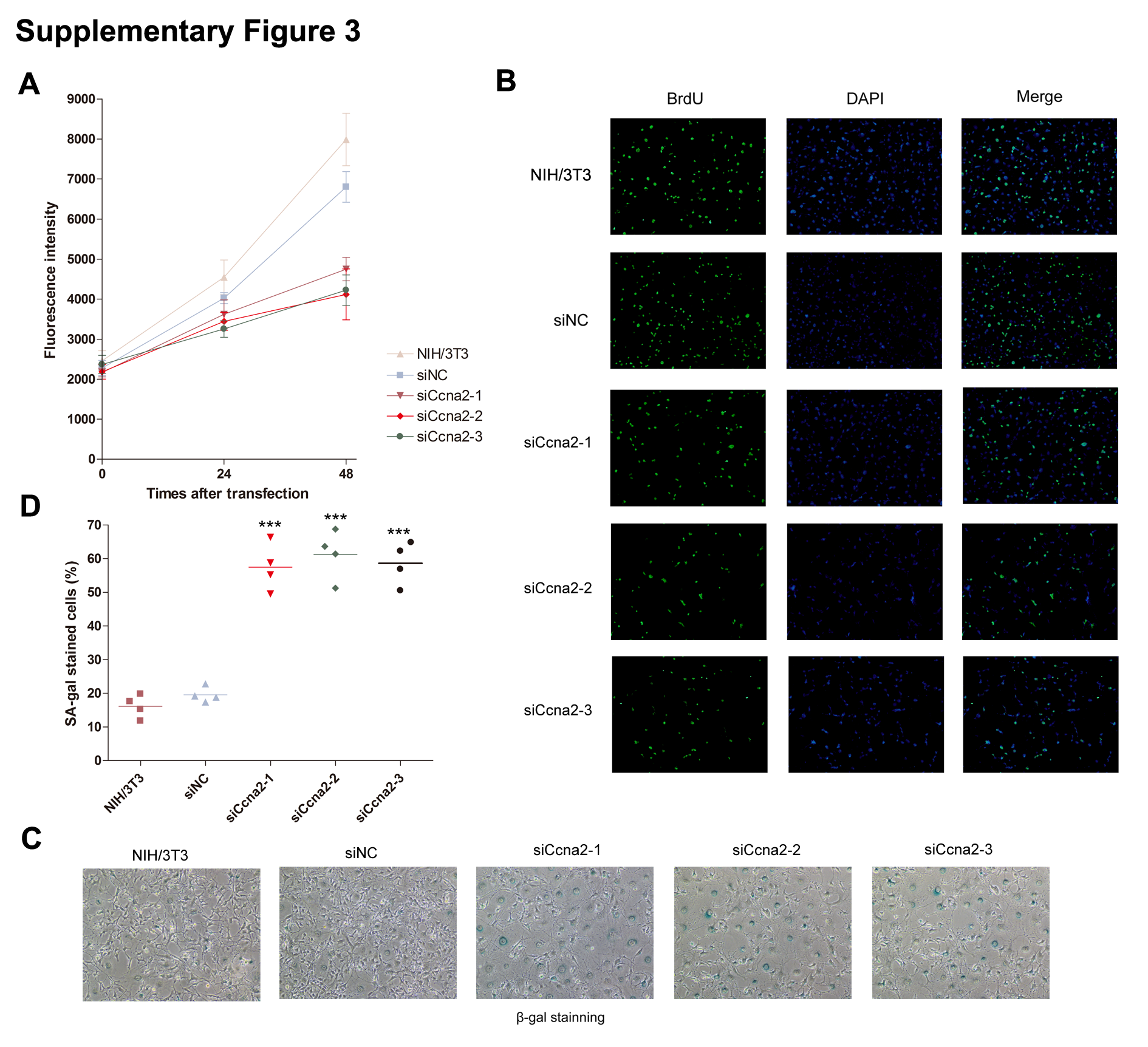

Supplement: Supplementary file 3 [file ACEL-18-e12918-s003.tif]

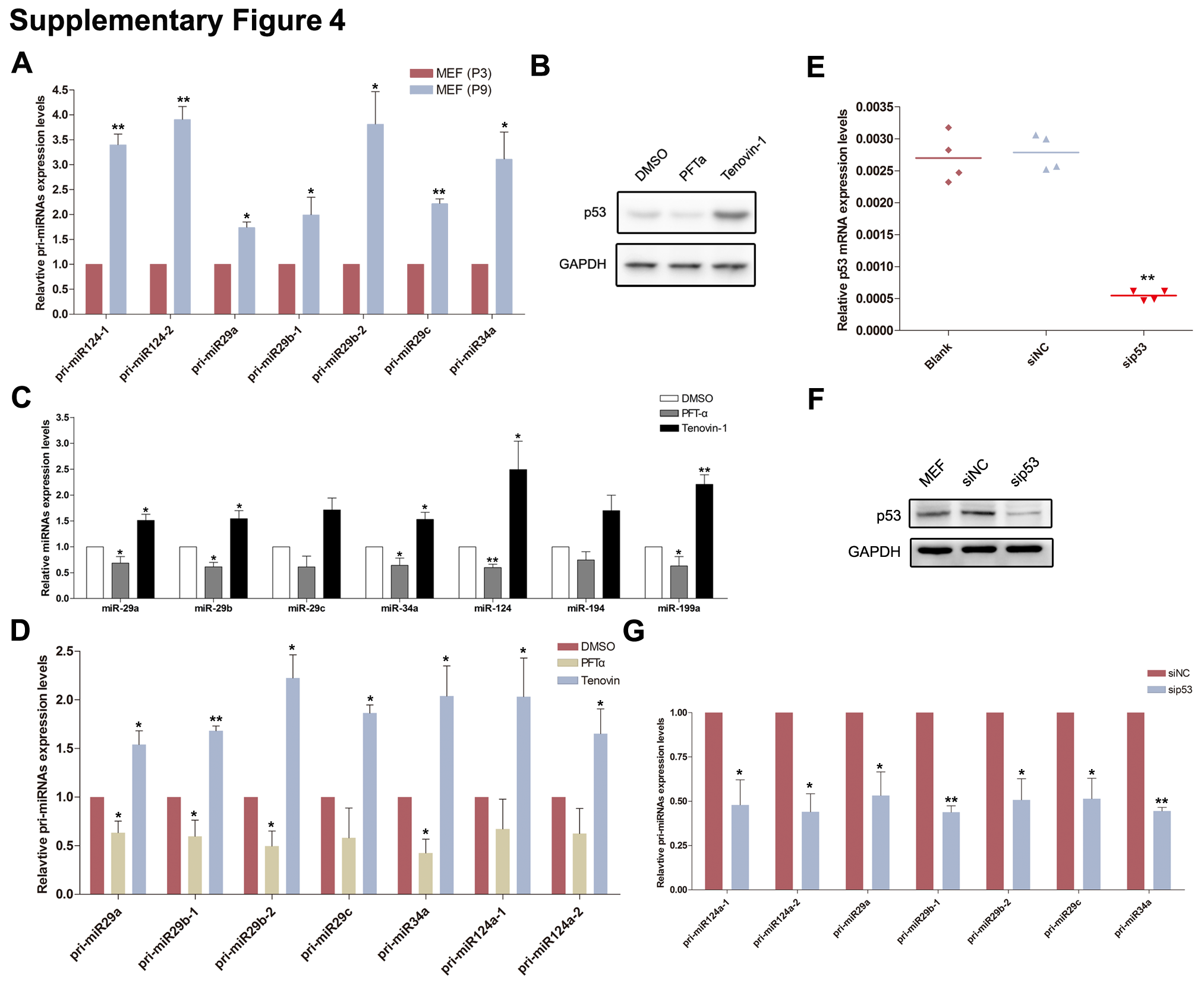

Supplement: Supplementary file 4 [file ACEL-18-e12918-s004.tif]

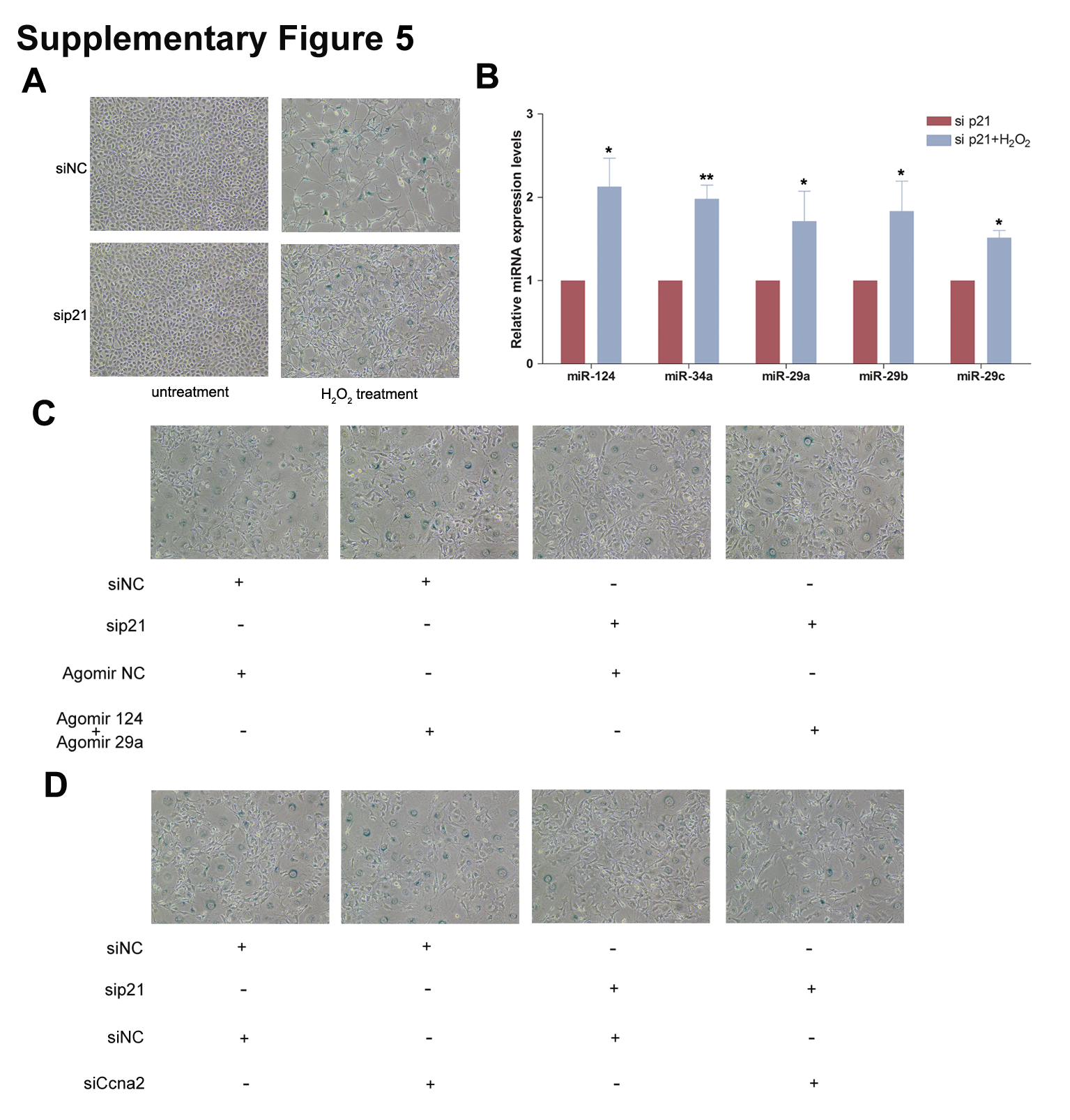

Supplement: Supplementary file 5 [file ACEL-18-e12918-s005.tif]

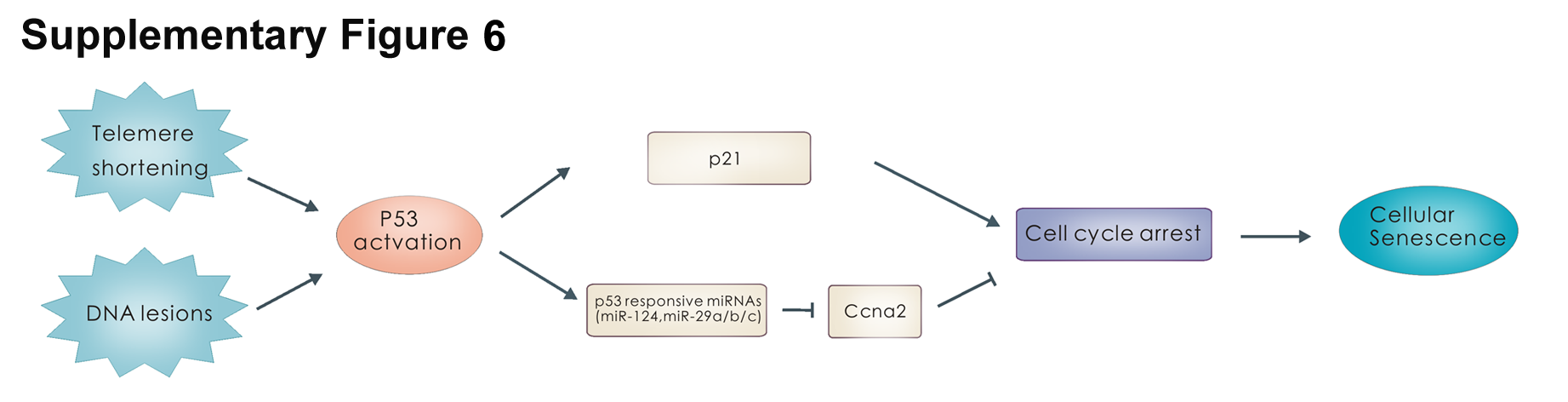

Supplement: Supplementary file 6 [file ACEL-18-e12918-s006.tif]
