# Supplementary material for: The p53/miRNAs/Ccna2 pathway serves as a novel regulator of cellular senescence: Complement of the canonical p53/p21 pathway
Source: Aging Cell. 2019 Mar 7;18(3):e12918. doi: 10.1111/acel.12918 (PMC6516184; doi:10.1111/acel.12918)
Supplement: Supplementary file 9 [file ACEL-18-e12918-s009.doc]

| **miRNA microarray** | | **mRNA microarray** | |
| --- | --- | --- | --- |
| **miRNA** | **Expression** | **Candidate Target gene** | **Expression** |
| **miR-124** | Up | Fanci, Peg3, Tyw3, E2f7, Acan, Clic6, Tmem169, Aurka, Wnt9b, **Ccna2**, Mboat2, Tcl1b2, Rnf122, Pgm5, Rasef | Down |
| miR-34a | Up | Iqgap3, Ndrg4, Mogat2, Adamts15, Ppard, Mest, Rasef, Sptlc3, Top2a, Dab1, Cdca8, Nuf2, Il31ra, Tyw3, Pgm5, Grhl1, Elf5, Eln, Corin, **Ccna2**, Taf1 | Down |
| **miR-29a/b/c** | Up | Mest, Eln, Ndrg4, Depdc1a, E2f7, Xpnpep2, **Ccna2**, Ppard, Wnt9b, Dab1, Fga, Elf5, Wnt11, Caps2, Cdca8, Fanci, Anapc7, Arntl, Acan, Pgm5 | Down |

**Supplementary Table 3. The predicted miRNA-target pairs**
