# Supplementary material for: The p53/miRNAs/Ccna2 pathway serves as a novel regulator of cellular senescence: Complement of the canonical p53/p21 pathway
Source: Aging Cell. 2019 Mar 7;18(3):e12918. doi: 10.1111/acel.12918 (PMC6516184; doi:10.1111/acel.12918)
Supplement: Supplementary file 10 [file ACEL-18-e12918-s010.doc]

**Supplementary Table 4.** **Sequences of primers for qPCR and 3’UTR cloning**

| **Name** | **Sense Primer (5'-3')** | **Antisense Primer (5'-3')** |
| --- | --- | --- |
| **Primers for real-time qPCR** | | |
| Ccna2 | GCCTTCACCATTCATGTGGAT | TTGCTCCGGGTAAAGAGACAG |
| Cdk1 | AGATCAGACTTGAAAGCGAGGA | GCAGGCTGACTATATTTGGATGT |
| E2f7 | AGGATGCGTTCGTGAACTCC | TGACAAGGGGTAGCTCGGATA |
| Ccnb1 | CTTGCAGTGAGTGACGTAGAC | CCAGTTGTCGGAGATAAGCATAG |
| Bub1 | ACAACCAGGGAATTGGAACCA | TTGGAATAGCCTGTATTGTTGCT |
| Bub1b | GGCTGAAGAATACGAAGCTAGAG | AGCCTTGCGTTCAATCCCTT |
| Aukra | CTGGATGCTGCAAACGGATAG | CGCTGGGAGTTAGAAGGACAC |
| Timp1 | CGAGACCACCTTATACCAGCG | ATGACTGGGGTGTAGGCGTA |
| Vcam1 | TTCGGTTGTTCTGACGTGTG | TACCACCCCATTGAGGGGAC |
| Dll1 | GACCTCGCAACAGAAAACCCA | TTCTCCGTAGTAGTGCTCGTC |
| Mmp9 | GGACCCGAAGCGGACATTG | CGTCGTCGAAATGGGCATCT |
| β-Actin | AGATGACCCAGATCATGTTTGAG | AGGGCATACCCCTCGTAGAT |
|  |  |  |
| **Primers for 3'UTR cloning** | | |
| Ccna2-3’UTR | AGTCTCGAGCTCAACCCACCAGAGACACT | AGTGCGGCCGCGCATTCTGGCCTACATGTCC |
