# Supplementary material for: The p53/miRNAs/Ccna2 pathway serves as a novel regulator of cellular senescence: Complement of the canonical p53/p21 pathway
Source: Aging Cell. 2019 Mar 7;18(3):e12918. doi: 10.1111/acel.12918 (PMC6516184; doi:10.1111/acel.12918)
Supplement: Supplementary file 11 [file ACEL-18-e12918-s011.doc]

**Supplementary Table 5.** **Sequences of the RNA oligoribonucleotides**

| **Name** | **Sense strand (5'-3')** |
| --- | --- |
| Agomir 124 | UAAGGCACGCGGUGAAUGCC |
| Agomir 34a | UGGCAGUGUCUUAGCUGGUUGU |
| Agomir 29a | UAGCACCAUCUGAAAUCGGUUA |
| Agomir NC | UUCUCCGAACGUGUCACGUTT |
| Antagomir 124 | GGCAUUCACCGCGUGCCUUA |
| Antagomir 34a | ACAACCAGCUAAGACACUGCCA |
| Antagomir 29a | UAACCGAUUUCAGAUGGUGCUA |
| Antagomir NC | CAGUACUUUUGUGUAGUACAA |
