# Supplementary material for: The p53/miRNAs/Ccna2 pathway serves as a novel regulator of cellular senescence: Complement of the canonical p53/p21 pathway
Source: Aging Cell. 2019 Mar 7;18(3):e12918. doi: 10.1111/acel.12918 (PMC6516184; doi:10.1111/acel.12918)
Supplement: Supplementary file 13 [file ACEL-18-e12918-s013.doc]

**Supplementary Figure 1.** miR-124, miR-34a and miR-29a/b/c were significantly up-regulated in H_2_O_2_ induced senescent NIH/3T3 cells. (A) Representative photographs of SA-β-gal staining of P3 MEF and P9 MEF. (B) Representative photographs of SA-β-gal staining of NIH/3T3 cells and H_2_O_2_ induced senescent NIH/3T3 cells. (C) Real-time qPCR was performed to monitor the miRNA expression levels in NIH/3T3 cells and H_2_O_2_ induced senescent NIH/3T3 cells. Columns, mean of four independent experiments (n=3); bars, SEM. *, *P* < 0.05; **, *P* < 0.01, comparison between two groups as indicated. (D) The expression levels of miR-124, miR-29a and miR-34a in serum starvation (0.1% FBS) induced quiescent MEFs. Columns, mean of four independent experiments (n=4); bars, SEM. *, *P* < 0.05, compared to MEFs (P3). (E) and (F) The mRNA and protein expression levels of Ccna2 in quiescent MEFs.

**Supplementary Figure 2.** miR-124 and miR-29a promoted H_2_O_2_-induced HUVECs senescence. (A) and (B) Representative photographs (A) and the average (B) of SA-β-gal staining of HUVECs transfected with Agomirs (Agomir NC, Agomir 124 and Agomir 29a) or Antagomirs (AntagomirNC, Antagomir 124 and Antagomir 29a). *, *P* < 0.05; **, *P* < 0.01, comparison between two groups as indicated. (C) p16 protein levels were evaluated using western blot in HUVECs transfected with Agomirs (Agomir NC, Agomir 124 and Agomir 29a) or Antagomirs (AntagomirNC, Antagomir 124 and Antagomir 29a).

**Supplementary Figure 3.** Knock-down of Ccna2 significantly suppressed the NIH/3T3 cells proliferation. (A) Effect of siCcna2 on cell viability of NIH/3T3 cells transfected with siNC and siCcna2-1/2/3. (B) Representative images of cells stained with DAPI (blue fluorescence) and BrdU (green fluorescence) in siNC and siCcna2-1/2/3 transfected NIH/3T3 cells.(C) Representative photographs of SA-β-gal staining of NIH/3T3 cells transfected with siNC and siCcna2-1/2/3. (D) The average SA-β-gal staining NIH/3T3 cells transfected with siNC and siCcna2-1/2/3. ***, *P* < 0.001, compared with the blank and siNC-transfected NIH/3T3 cells.

**Supplementary Figure 4.** Verification the correlation between p53 and p53 responsive miRNAs. (A) The pri-miRNAs expression levels of miR-124, miR-29 and miR-34a. Columns, mean of four independent experiments (n=4); bars, SEM. *, *P* < 0.05; **, *P* < 0.01, comparison between two groups as indicated. (B) Western blot was performed to monitor the p53 expression levels in MEFs treated with inhibitor (pifithrin-α, PFTα) or activator (Tenovin-1). (C) and (D) Real-time qPCR was performed to evaluate the mature (C) and pri-miRNA (D) expression levels of miRNAs in PFTα or Tenovin-1 treated MEF cells. Columns, mean of four independent experiments (n=4); bars, SEM. *, *P* < 0.05; **, *P* < 0.01, compared to the DMSO treated cells. (E) and (F) Real-time qPCR and western blot were performed to detect the p53 mRNA and protein levels in MEFs transfect with siNC and sip53. (G) The pri-miRNA expression levels of miRNAs in siNC and sip53 transfected MEF cells. Columns, mean of four independent experiments (n=4); bars, SEM. *, *P* < 0.05; **, *P* < 0.01, compared to the siNC transfected MEF cells.

**Supplementary Figure 5.** (A) Representative photographs of SA-β-gal staining of NIH/3T3 cells transfected with siNC and sip21 (sip21-3) with or without H_2_O_2_ treatment. (B) Real-time qPCR was performed to detect the miRNA expression levels in NIH/3T3 cells transfected with sip21 with or without H_2_O_2_ treatment. Columns, mean of four independent experiments (n=3); bars, SEM. *, *P* < 0.05; **, *P* < 0.01, comparison between two groups as indicated. (C) Representative photographs of SA-β-gal staining of NIH/3T3 cells co-transfected with siNC or sip21, and Agomir 124/Agomir 29a or Agomir NC. (G) Representative photographs of SA-β-gal staining of NIH/3T3 cells co-transfected with with siNC or sip21, and siNC or siCcna2.

**Supplementary Figure 6.** Schematic diagram of the p53/p53 responsive miRNA/Ccna2 pathway and the canonical p53/p21 pathway.
